# Seed Oil Quality of *Brassica napus* and *Brassica rapa* Germplasm from Northwestern Spain

**DOI:** 10.3390/foods8080292

**Published:** 2019-07-27

**Authors:** Elena Cartea, Antonio De Haro-Bailón, Guillermo Padilla, Sara Obregón-Cano, Mercedes del Rio-Celestino, Amando Ordás

**Affiliations:** 1 Plant Genetic and Breeding Department, Biological Mission of Galicia (CSIC), Apartado 28, E-36080 Pontevedra, Spain; 2 Plant Breeding Department, Institute for Sustainable Agriculture (CSIC), Alameda del Obispo s/n, 14080 Córdoba, Spain; 3 Bioinformatics and Biostatistics Service, Biological Reseach Center (CSIC), Ramiro de Maeztu 9, 28040 Madrid, Spain; 4 Agri-food Laboratory, (CAPDER), Avda Menéndez Pidal, s/n, 14080 Córdoba, Spain

**Keywords:** *Brassica napus*, *Brassica rapa*, fatty acid composition, germplasm, oil content

## Abstract

The seed oil content and the fatty acid composition of a germplasm collection of *Brassica napus* and *Brassica rapa* currently grown in Galicia (northwestern Spain) were evaluated in order to identify potentially interesting genotypes and to assess their suitability as oilseed crops for either edible or industrial purposes. The seeds of the *B. rapa* landraces had higher oil content (mean 47.3%) than those of *B. napus* (mean 42.8%). The landraces of both species showed a similar fatty acid profile (12% oleic acid, 13% linoleic acid, 8–9% linolenic acid, 8–9% eicosenoic acid, and 50–51% erucic acid). They were very high in erucic acid content, which is nutritionally undesirable in a vegetable oil, and very low in oleic and linoleic acid contents. Therefore, they could be used for industrial purposes but not as edible oil. The erucic acid content ranged from 42% to 54% of the total fatty acid composition with an average value of 50% in the *B. napus* landraces whereas in *B. rapa*, it ranged from 43% to 57%, with an average value of 51%. Considering the seed oil and the erucic acid content together, three varieties within the *B. napus* collection and two varieties within the *B. rapa* one seem to be the most promising genotypes for industrial purposes.

## 1. Introduction

*Brassica* oilseed crops have become the third most important source of edible vegetable oils in the world [1]. Although edible oils currently represent the largest market for *Brassica* oilseed crops, the prevalence of agricultural surpluses in many developed countries has focused attention toward the possible industrial use of *Brassica* seed oils. The usefulness and quality characteristics of seed oils are determined by the proportion of its main constituent fatty acids [2,3,4]. Consequently, one of the most important objectives in *Brassica* breeding is the genetic modification of seed oil by maximizing the proportion of specific fatty acids [5,6,7,8].

*Brassica* oil is considered beneficial from a health point of view. It contains linoleic acid, which is desirable for nutritional purposes, and oleic acid, whose thermostability makes it desirable for cooking oil [9]. High oleic acid oil tastes better and may also have health benefits. The oxidative stability of this fatty acid also makes it suitable for some industrial applications [10]. Nevertheless, *Brassica* oil is characterised by significant amounts of erucic acid (about 50% of the total fatty acids), which is absent in any other commercial plant oils [11,12]. Erucic acid (cis-13-docosenoic acid, 22:1) has 22 carbon atoms with one double bond at the cis-13 position of the carbon chain. Oil with high erucic acid content has anti-nutritional properties but is suitable for some industrial applications, such as anti-blocking agents in polyethylene films, adhesives in printing, and anticorrosive materials in the steel sheet metal industry [9,13,14]. They may also be used in the manufacture of cosmetics products through the synthesis of waxes that could be used as a jojoba oil substitute [15,16]. The oleochemical industry demands oils with high levels of erucic, behenic, and arachidic fatty acids. In recent decades, oilseed *Brassica* crops have also gained attention not only as a source of edible oils but also as a source of bio-fuel and industrial feed-stock. These genera have regained interest for use in cosmetics, in the emollient industry for lubricant, and for adhesive and biodegradable plastic products [4,17]. A medicinal application has also been found for erucic acid, administrated in therapeutic doses, to treat adrenoleukodystrophy (X-ALD), a genetic disorder that damages the nervous system and is associated with the accumulation of very long chain fatty acids [18,19]. Therefore, the fatty acid compositions of rapeseed oils have been modified according to specific objectives through conventional and molecular breeding [8]. The production of biodiesel has offered new opportunities and also lead to changes in the orientation of rapeseed consumption and utilization. Moreover, the emerging emphasis on renewable energy, chemical feed stocks, industrial oils, and the steadily growing bioeconomy will provide significant growth opportunities for industrial *Brassica* oils.

The development of commercial varieties free of erucic acid and with very high erucic acid content are breeding objectives in *Brassica* oilseed crops [7,13,17,20]. Agronomically acceptable cultivars producing low erucic acid oils were first available in the *B. napus* cultivar ‘Oro’ in 1968 and in the *B. rapa* cultivar ‘Span’ in 1971 [17,21]. The term ‘canola oil’ describes the oil profile of the current *B. napus* and *B. rapa* cultivars used for the production of edible oil with very low erucic acid content.

The genus *Brassica* encompasses very diverse types of plants grown as vegetables, fodder, and sources of oils and condiments. The species *B. napus*, *B. rapa*, *B. juncea*, and *B*. *carinata*, generally known as rapeseed, form the oilseed group [4,17]. Within the *B. rapa* and *B. napus* species there are also vegetable crops used for human nutrition, such as turnip, turnip tops or turnip greens (*B. rapa* ssp. *rapa*) and leaf rape (*B. napus* var. *pabularia*), which are widely grown in Galicia (northwestern Spain). *B*. *napus* var. *pabularia* crops grown in Galicia are known as ‘nabicol’ [22]. These populations are the result of mass selection carried out by growers who have been using them as leafy greens for many years, since the use of commercial varieties in this area is not common yet. The agronomic performance, morphological attributes, and leaf nutritional value of the *Brassica* germplasm grown in northwestern Spain have been extensively studied in *B. napus* [23,24]) and *B. rapa* [25,26,27]. The potential use of the genetic diversity existing in the *B. napus* landraces was described by Cartea et al. [21]. De Haro et al. [28] reported a preliminary work about the seed oil composition for a set of *Brassica* landraces from northwestern Spain and found that its accessions had very high erucic and very low oleic and linolenic acid contents. The suitability of other *Brassica* species as sources of new potential oilseed crops has been reported for *B. carinata* [29,30,31,32] and *B. juncea* [33,34].

The objectives of the present work were to evaluate the seed quality (the seed oil content and fatty acid composition) of a germplasm collection of *B. napus* and *B. rapa*, to identify potentially interesting genotypes, and to assess its suitability as oilseed crops for edible or industrial purposes.

## 2. Materials and Methods

### 2.1. Plant Material

A set of 41 accessions of ‘nabicol’ (*B. napus* ssp. *pabularia*) comprising 38 landraces and 3 commercial varieties, and a set of 169 accessions of turnip, turnip tops, and turnip greens (*B. rapa* ssp. *rapa*), including 162 landraces and 7 commercial varieties from the germplasm collection of the Biological Mission of Galicia (Misión Biológica de Galicia, MBG, Pontevedra), in Spain were analysed for total seed oil content and fatty acid composition. This material is stored and maintained as an active collection at the MBG.

**Figure 1:**
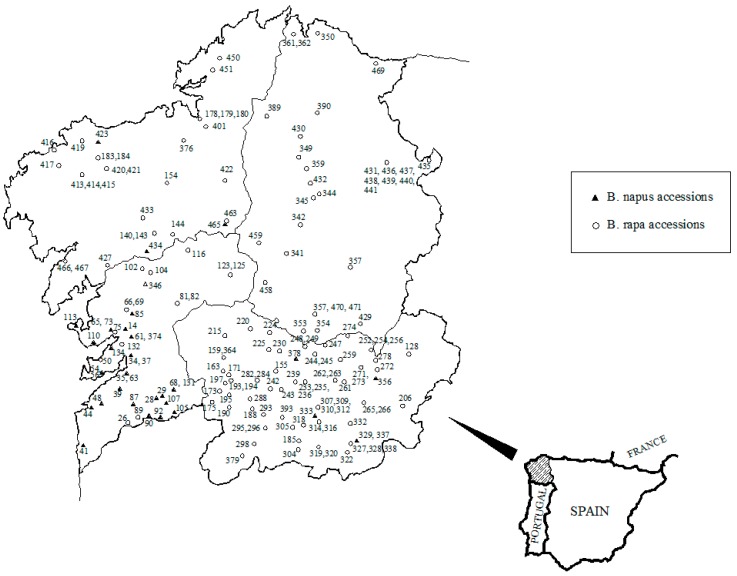
Map of Galicia (northwestern Spain) with the geographical distribution of the *B. rapa* and *B. napus* landraces evaluated in this study.

These accessions represent the genetic variability of the *B. rapa* and *B. napus* germplasm currently grown in Galicia. The landraces were collected directly from growers at different sites throughout northwestern Spain from the eighties to present, and some of them have been propagated under isolation at the MBG in different years. Seed samples from different genotypes were taken from accessions kept at the germplasm bank at the MBG under the same low temperature and seed moisture conditions. Varieties were multiplied over several years, but always in the same location, with the same experimental plot, and under the same growing conditions. Due to the high number of genotypes evaluated in this study, it would have been impossible to multiply all the varieties in the same year. The geographical distribution of the *B. rapa* and *B. napus* landraces is shown in Figure 1. The number of landraces comprising the *B. napus* collection was lower than the *B. rapa* ones since the growing region of *B. napus* is restricted to the coastal area of southern Galicia and to areas near the Portuguese border (Figure 1). Here, the crop is well adapted and common in the human diet [21]. Five accessions of *B. napus* were collected in inland areas, probably due to both human migration and the sale of seeds in local markets. All the *B. napus* landraces come from Galicia, Spain whereas the 3 *B. napus* commercial varieties were bought in Vila Real (North of Portugal), since commercial varieties of *B. napus* are not found in Galicia. All the *B. rapa* entries come from Galicia, Spain.

### 2.2. Lipid Analysis

Bulk samples of seeds of each accession (landraces and commercial varieties) were screened for oil content. The oil content of the seeds was determined by nuclear magnetic resonance (NMR) with an Oxford 4000 Analyzer (Oxford Analytical Instruments Ltd., Abingdon, OX, UK), following desiccation at 50 °C for 72 h. For fatty acid composition, ten seeds were randomly selected and individually analysed for each local and commercial variety. The content of seven major fatty acids present in the oil extracted from *Brassica* crops (palmitic, stearic, oleic, linoleic, linolenic, eicosenoic, and erucic), as well as the content of other minor fatty acids (arachidic, arachidonic, and behenic), was determined. In order to evaluate the fatty acid composition of seed samples, the lipids were extracted, transmethylated, and purified using the one-step method of Garcés and Mancha [35] with some modifications. Individual seed samples were heated at 80 °C for 2 h in MeOH: toluene: dimethoxypropane: H_2_SO_4_: heptane (33:14:2:1:50; by vol.), and, after cooling, the fatty acid methyl esters were recovered in the upper phase. The analysis of the fatty acid methyl esters composition was developed in a Perkin Elmer Autosystem gas-liquid chromatograph (Perkin-Elmer Corporation, Norwalk, USA) equipped with a flame ionization detector (FID) and a 2 m long column packed with 3% SP-2310/2% SP-2300 on a Chromosorb WAW (Supelco Incorporated, Bellefonte, USA). The gas chromatograph was programmed for an initial temperature of 190 °C for 10 min followed by an increase of 2 °C per min to 220 °C; this final temperature was maintained for a further 5 min. The injector and flame-ionization detector were held at 275 and 250 °C, respectively. The fatty acids were identified by comparison with known fatty acid methyl esters standards (F.A.M.E. Mix, CRM18920 Supelco and ME14-1KT Supelco, Sigma-Aldrich). The analyses were performed at the ‘Institute for Sustainable Agriculture (*Instituto de Agricultura Sostenible*, IAS), Spain.

Individual and combined analyses of variance were performed for each trait of seed composition, using the general lineal model (GLM) procedure of the SAS statistical package [36]. The accessions and the species were considered as fixed effects. Comparisons of means among populations and species were performed for each trait using Fisher’s protected least significant difference (LSD) at *p* = 0.05 [37].

## 3. Results and Discussion

### 3.1. Oil Content in the Brassica Collection

The oil content in the seeds of the *B. napus* landraces ranged from 29.1% (for MBG-BRS0423) to 50.1% (for MBG-BRS0044), with an average value of 42.5%. The oil content of the *B. rapa* landraces ranged from 31.4% (for MBG-BRS0285) to 56.3% (for MBG-BRS0245), with an average value of 47.3% (Table 1). The *Brassica rapa* genotypes were significantly higher in oil content than the *B. napus* ones (Table 1). This result agrees with Mandal et al. [38], who found that seed oil content was higher in a collection of *B. rapa* (about 42%) than in a collection of *B. napus* (about 35%). The seed oil content from the *B. napus* landraces was significantly lower than that from the commercial varieties, whereas no significant differences were found between the landraces and the commercial varieties in *B. rapa*. The genotypes evaluated in this work showed values for oil content similar to those found on cultivars of the major *Brassica* oilseed crops (*B. napus, B. rapa, B. carinata,* and *B. juncea*), with an average oil content between 45% and 50% [30,39], even though the *Brassica* germplasm from northwestern Spain is not grown as an oilseed crop. Since only one datum per population was obtained, mean oil content comparisons among populations are not reported. Despite this fact, the highest seed oil content (more than 47%) in the *B. napus* collection was found for landraces MBG-BRS0044, MBG-BRS0087, MBG-BRS0329, MBG-BRS0434, and MBG-BRS0105, along with the commercial variety, MBG-BRS0373. For the *B*. *rapa* collection, landraces MBG-BRS0245, MBG-BRS0236, MBG-BRS0125, MBG-BRS0249, and MBG-BRS0139 had the highest levels.

### 3.2. Fatty Acid Composition in the Brassica Collection

Significant differences among the *B. napus* landraces were found for all the fatty acids analysed, whereas the commercial varieties of this species were not significantly different for linoleic and erucic acid contents (Table 2). The fatty acid profile observed between the landraces and the commercial varieties of *B. napus* was different. The average erucic acid content was considerably higher in the *B. napus* landraces compared to the commercial seeds (Table 3). The commercial varieties had zero erucic acid and followed the typical profile of canola varieties even though they are commonly used as vegetable crops but not as oilseed crops. The collection of the *B. napus* evaluated includes all the germplasm currently grown in Galicia, which means that in this region, only the *pabularia* type is grown, and *B. napus* is not used for oil production. The significant differences between the *B. napus* landraces and the commercial varieties do not mean that the commercial *pabularia* breeding is based on modern zero erucic varieties of *B. napus*. Since commercial varieties of *B. napus* are not common in Galicia, the seeds used in this study came from Portugal, where they were bought as ‘couve-nabiça’ (Portuguese *B. napus* landrace), but they are probably zero erucic rapeseed varieties.

Within the *B. rapa* collection, the landraces and the commercial varieties were significantly different for all the fatty acids. The seeds of the *B. rapa* commercial varieties, which included crops of turnips, turnip greens, and turnip tops, were significantly different in erucic acid content (Table 2), although their values were significantly lower than those found in most landraces (Table 3). Despite this fact, the genotypes of both types of germplasm (landraces and commercial varieties) showed a similar fatty acid profile (Table 3).

Significant differences were found between the *B. napus* and *B. rapa* landraces for all the fatty acids, except for oleic and erucic acids (Table 2). The seeds of the *B. rapa* landraces were higher than the *B. napus* ones for stearic, linoleic, linolenic, and eicosenoic acids, while the *B. napus* landraces were only higher for palmitic acid content (Table 3).

The landraces of both species were high in erucic acid and low in oleic, linoleic, linolenic, and eicosenoic acids; palmitic and stearic acids were minor, and arachidic, arachidonic, and behenic acid were negligible (Table 3). The fatty acid profile of the oil contained in the *B. napus* seeds (12% oleic acid, 13% linoleic acid, 8% linolenic acid, 8% eicosenoic acid, and 50% erucic acid) was very similar to that found in the seeds of *B. rapa* (12% oleic acid, 13% linoleic acid, 9% linolenic acid, 9% eicosenoic acid, and 51% erucic acid) (Table 3). Both fatty acid profiles contrast with the typical profile of canola oil, which can be represented as 61% oleic acid, 21% linoleic acid, 11% linolenic acid, and no erucic acid [4,8,10].

Since erucic acid was the major fatty acid found, and because it is a trait of large interest for plant breeding, most discussion will be focused on this fatty acid. The erucic acid content ranged from 42% to 54% of the total fatty acid composition, with an average value of 50% in the *B. napus* landraces and from 43% to 57% of the total fatty acid composition in the *B. rapa* landraces, with an average value of 51% (Table 3). Similar values for erucic acid content were found by Sharafi et al. (2015) [12] for three rapeseed varieties and for four entries of *B. rapa*, although crops were different from those evaluated in this study. The lowest content of erucic acid was found within the *B. napus* species in seeds of MBG-BRS0333 (about 42% of the total fatty acids) and within the *B. rapa* species in seeds of MBG-BRS0379 (about 43% of the total fatty acids). Both values are still very high, and, therefore, vegetable oil of the genotypes evaluated should be considered unsuitable for edible oil production. On the other hand, oils with high erucic acid content are desirable for industrial purposes. For both species, the landraces with high erucic acid content are included in Table 4. The highest erucic acid composition, more than 53% of the total fatty acids, was found in *B. napus* landraces MBG-BRS0329, MBG-BRS0041, MBG-BRS0048, and MBG-BRS0105 (Table 4). *B. rapa* landraces MBG-BRS0235, MBG-BRS0416, MBG-BRS0202, MBG-BRS0139, MBG-BRS0190, and MBG-BRS0239 showed the highest erucic acid composition—more than 55% of the total fatty acids (Table 4). Considering their high oil content and high erucic acid content together, genotypes MBG-BRS0329, MBG-BRS0434, and MBG-BRS0105 within the *B. napus* collection, and MBG-BRS0139 and MBG-BRS0101 within the *B. rapa* collection, offer interesting prospects for future industrial applications. The *Brassica* landraces grown in Galicia have been traditionally improved by growers for vegetable use but not for their use as oil sources. Nowadays, efforts to develop low erucic acid genotypes of both *B. napus* var. *pabularia* and *B*. *rapa* ssp. *rapa* have not been undertaken because the main usage of these crops is for leaf consumption.

High erucic acid contents in the seed oil of different *Brassica* crops have been previously reported by several authors. De Haro et al. [30] found high values of erucic acid in the seed oil of some accessions analysed from these same collections, between 43.3 and 57.2% in *B. napus*, and between 42.7 and 53.2% in *B. rapa*. Velasco et al. [40] reported high levels of erucic acid (about 40% of the total fatty acids) in a *B. napus* collection comprising 25 accessions, where four were *B. napus* crops. High erucic acid levels were also found by Velasco et al. [40] in a set of 72 genotypes of *B. rapa* (about 45% of the total fatty acids), where five were *B. rapa* ssp. *rapa*, although the average erucic acid content for these five accessions was lower (37.6%) than the values found in the present work. Mandal et al. [38] evaluated the fatty acid content of several cruciferous species and reported erucic acid contents of 41% in genotypes of *B. napus,* 47.4% in *B. rapa* ssp. *dichotoma*, and 51.6% in *B. rapa* ssp. *trilocularis*. Rapeseed oil genotypes with higher proportions of erucic acid than the levels found in traditional cultivars (about 50%) are sought by breeders for use in well-known industrial products [4,7,13,20]. In this way, the above-mentioned populations of *B. napus* and *B. rapa* could be used in industrial processes but they are not appropriate for edible uses.

In general, low intra-population variability was observed for the fatty acid composition of the seed oil. However, some landraces displayed an important variation among the ten seeds individually analysed for erucic acid content. Some of them had seeds with either decreased or increased values of that fatty acid. In the *B. napus* collection, three individual seeds of MBG-BRS0337 were found to have less than 35% erucic acid content (27.2%, 31.7%, and 33.1%). On the other hand, one individual seed of both MBG-BRS0105 and MBG-BRS0329 was found to have more than 56% of erucic acid content. In the *B. rapa* collection one individual seed of MBG-BRS0431 and two seeds of MBG-BRS0463 had low values for erucic acid content, (32.3%, 33.9%, and 34.1%, respectively), whereas three landraces, MBG-BRS0195, MBG-BRS0224, and MBG-BRS0231 had individual seeds with values higher than 60% of erucic acid content. The decreased content of erucic acid could be due to an unintended cross with a zero or low erucic commercial variety. However, in order to avoid the problem of pollen contamination, the seeds analyzed were directly obtained from growers or from multiplications made in isolation cages at the MBG. The decreased or increased levels of erucic acid content found in some seeds could presumably correspond to heterozygous seeds for that fatty acid. It would be interesting to analyse more seeds from the above-mentioned populations using the half-seed method described for other oilseed crops [41] and to select genotypes in segregating populations with lower or higher erucic acid content than that observed in their respective landraces.

There are numerous studies on the nutritional and industrial value of the seed oil of Brassica oilseed crops, but there are no previous studies on the potential of seed oil in these vegetable Brassica crops. Therefore, the present work provides relevant information and discussion on the potential of the oil of the seeds of two horticultural crops of Brassica, nabicol and turnip greens, for food or industrial purposes. The reported results offer the first insights into the variability of the current gene pool of the *B. rapa* and *B. napus* varieties grown in Galicia. These valuable genetic resources will certainly be studied regarding for important traits. The content of glucosinolates should be taken into account, as glucosinolates are important in modern rapeseed breeding and are probably very high in the landraces of both species. As a conclusion, the germplasm evaluated in this work displayed variability in the fatty acid composition of its seed oil. Some accessions of both species could be further used as sources of oil for industrial purposes because their seeds were high in erucic acid content and low in oleic, linoleic, and linolenic acid content. Further research will be needed for some accessions having seeds with reduced or increased values of erucic acid content, in order to select valuable genotypes that could be used for both nutritional and industrial applications.

## Figures and Tables

**Table 1:** Mean, range (minimum and maximum values), and standard deviation of oil content in the seeds of *B. napus* and *B. rapa* varieties from northwestern Spain.

	Oil Content (%)			
Accessions	N°	Mean	Range	Standard Deviation
*Brassica napus* collection	41	42.80		
Landraces	38	42.46	(29.06–50.11)	3.88
Commercial varieties	3	47.13	(46.35–48.50)	1.19
LSD (5%)		4.60		
*Brassica rapa* collection	169	47.27		
Landraces	162	47.22	(31.38–56.34)	4.31
Commercial varieties	7	48.59	(46.04–52.85)	2.09
LSD (5%)		3.24		
LSD (5%) between landraces ^1^		1.50		

**N°** = Number of accessions studied; LSD = least significative differenc; ^1^ Comparison between 38 *B. napus* and 162 *B. rapa* landraces.

**Table 2:** Mean squares of the analysis of variance for fatty acids in the *B. napus* and *B. rapa* varieties studied.

Sources of Variation	df	C16:0	C18:0	C18:1	C18:2	C18:3	C20:1	C22:1
*Brassica napus*	40	2.35 **	0.31 **	1754.28 **	56.91 **	11.24 **	49.73 **	1803.20 **
Landraces	37	0.83 **	0.08 **	70.15 **	15.50 **	9.41 **	15.11 **	85.53 **
Commercial var.	2	0.97 *	0.01 *	37.79 *	0.85	40.54 **	0.31 **	0.005
Between types	1	61.37 **	9.26 **	67,501.8 **	1700.9 **	20.20 **	1409.30 **	68,961.78 **
*Brassica rapa*	168	2.21 **	0.41 **	42.34 **	8.73 **	8.64 **	13.53 **	67.21 **
Landraces	161	2.27 **	0.42 **	40.37 **	8.66 **	8.12 **	13.55 **	67.22 **
Commercial var.	6	0.72 **	0.11 **	32.68 **	11.05 **	12.47 **	9.35 **	35.36 **
Between types	1	2.64 *	0.32 *	418.70 **	6.32	70.32 **	37.41 **	257.05 **
Between landraces ^a^	1	43.67 **	0.66 **	7.78	11.34 **	4.57 *	6.78 *	16.21

*, ** significant at *p* < 0.05 and at *p* < 0.01, respectively. ^a^ Comparison between 38 *B. napus* and 162 *B. rapa* landraces. C16:0 = palmitic acid, C18:0 = stearic acid, C18:1 = oleic acid, C18:2 = linoleic acid, C18:3 = linolenic acid, C20:1 = eicosenoic acid, and C22:1 = erucic acid. df = degrees of freedom.

**Table 3:** Fatty acid composition ^a^ of the seed oil (mean, minimum and maximum values) of *B. napus* and *B. rapa* varieties from northwestern Spain.

Accessions	No.	C16:0	C18:0	C18:1	C18:2	C18:3	C20:1	C22:1
*Brassica napus*	41							
Landraces	38	2.95	0.58	12.37	12.72	8.26	8.27	49.83
		(2.30–3.50)	(0.40–0.77)	(7.85–18.73)	(7.94–15.74)	(6.51–10.87)	(6.23–10.58)	(42.35–54.09)
Commercial varieties	3	4.43	1.16	61.64	20.54	9.12	1.10	0.02
		(4.25–4.79)	(1.09–1.26)	(59.91–63.74)	(20.21–20.71)	(6.86–10.72)	(0.90–1.24)	(0.004–0.05)
LSD (5%) ^b^		0.172	0.054	1.265	0.610	0.510	0.646	1.463
*Brassica rapa*	169							
Landraces	162	1.75	0.72	11.86	13.33	8.65	8.73	50.56
		(0.66–2.57)	(0.25–1.28)	(7.56–18.53)	(10.91–16.56)	(5.25–10.84)	(4.72–11.95)	(42.75–56.96)
Commercial varieties	7	1.95	0.79	14.36	13.02	7.63	9.48	48.60
		(1.57–2.37)	(0.59–0.93)	(11.75–16.64)	(11.33–14.27)	(6.23–9.33)	(8.30–11.07)	(46.03–51.39)
LSD (5%) ^b^		0.181	0.067	0.709	-	0.334	0.442	0.951
LSD (5%) between landraces ^c^	0.159	0.070	-	0.355	0.325	0.419	-	

^a^ These values are the means of ten single seeds, expressed as % of the total fatty acids. ^b^ Comparison between the landraces and the commercial varieties of each species.^c^ Comparison between 38 *B. napus* and 162 *B. rapa* landraces. C16:0 = palmitic acid, C18:0 = stearic acid, C18:1 = oleic acid, C18:2 = linoleic acid, C18:3 = linolenic acid, C20:1 = eicosenoic acid, and C22:1 = erucic acid. **N°** = Number of accessions studied; LSD = least significative difference.

**Table 4:** Seed oil content and fatty acid composition ^a^ (major fatty acids) of *B. napus* and *B. rapa* landraces with the highest seed oil content and the highest erucic acid content.

Landraces	Oil Content	C16:0	C18:0	C18:1	C18:2	C18:3	C20:1	C22:1
*Brassica napus*								
MBG-BRS0329	47.47	2.3 ± 0.1	0.6 ± 0.1	16.5 ± 2.0	7.9 ± 1.0	6.6 ± 0.7	8.1 ± 1.6	54.1 ± 2.9
MBG-BRS0041	45.31	2.6 ± 0.1	0.5 ± 0.1	9.7 ± 1.4	12.0 ± 0.6	9.7 ± 0.7	6.4 ± 0.8	53.8 ± 1.2
MBG-BRS0048	42.88	2.9 ± 0.3	0.6 ± 0.1	9.9 ± 1.5	12.6 ± 0.7	8.1 ± 1.0	6.6 ± 1.1	53.8 ± 1.8
MBG-BRS0105	46.61	2.6 ± 0.2	0.5 ± 0.1	10.0 ± 1.4	12.2 ± 0.7	8.9 ± 0.5	7.2 ± 1.6	53.8 ± 2.6
MBG-BRS0465	42.55	2.7 ± 0.2	0.6 ± 0.1	9.7 ± 0.9	11.9 ± 0.5	9.8 ± 0.4	6.8 ± 0.9	52.9 ± 1.8
MBG-BRS0065	39.46	2.9 ± 0.3	0.6 ± 0.1	9.0 ± 1.2	13.9 ± 0.5	8.4 ± 0.5	7.0 ± 1.1	52.6 ± 1.9
MBG-BRS0028	40.77	2.8 ± 0.3	0.6 ± 0.1	9.7 ± 2.2	13.8 ± 0.9	13.8 ± 0.9	7.1 ± 1.7	52.5 ± 2.1
MBG-BRS0037	43.09	3.3 ± 1.5	0.5 ± 0.2	11.0 ± 2.1	12.5 ± 1.3	8.8 ± 1.2	6.3 ± 1.0	52.4 ± 2.5
MBG-BRS0063	40.01	3.1 ± 0.2	0.5 ± 0.1	10.1 ± 1.0	12.3 ± 0.8	8.3 ± 0.3	7.1 ± 0.6	52.2 ± 2.4
MBG-BRS0014	45.16	2.7 ± 0.2	0.6 ± 0.2	11.1 ± 2.0	12.1 ± 0.6	9.2 ± 0.9	7.7 ± 1.5	51.8 ± 2.5
MBG-BRS0434	47.30	2.7 ± 0.2	0.5 ± 0.1	10.8 ± 1.8	11.9 ± 0.7	9.4 ± 0.9	8.0 ± 1.5	51.8 ± 3.1
MBG-BRS0054	43.94	3.0 ± 0.1	0.5 ± 0.1	7.9 ± 1.1	13.9 ± 0.8	10.9 ± 0.6	6.2 ± 0.7	51.7 ± 1.9
MBG-BRS0374	45.48	3.0 ± 0.2	0.6 ± 0.1	11.8 ± 1.3	12.6 ± 0.6	8.1 ± 0.5	8.1 ± 1.1	51.5 ± 2.4
MBG-BRS0068	46.18	3.1 ± 0.3	0.6 ± 0.1	10.0 ± 1.6	13.1 ± 0.7	8.7 ± 0.5	7.7 ± 1.0	51.4 ± 2.9
*Brassica rapa*								
MBG-BRS0235	46.35	0 ^b^	0.1 ± 0.2	10.0 ± 1.4	14.6 ± 1.5	9.5 ± 0.8	6.0 ± 0.9	57.0 ± 1.6
MBG-BRS0416	46.63	1.6 ± 0.6	0.7 ± 0.2	8.8 ± 1.6	13.7 ± 0.8	9.0 ± 1.1	6.3 ± 1.8	55.7 ± 3.0
MBG-BRS0202	42.92	2.3 ± 0.5	0.9 ± 0.2	7.6 ± 1.7	12.7 ± 0.9	10.8 ± 1.1	4.7 ± 1.0	55.3 ± 2.6
MBG-BRS0139	54.34	1.3 ± 0.5	0.8 ± 0.1	9.9 ± 2.5	11.8 ± 0.8	9.1 ± 1.3	7.5 ± 1.8	54.9 ± 3.0
MBG-BRS0190	42.14	1.2 ± 0.5	0.6 ± 0.2	8.2 ± 1.1	13.8 ± 1.3	9.7 ± 1.3	6.7 ± 0.8	54.7 ± 1.5
MBG-BRS0239	47.97	0 ^b^	0.1 ± 0.1	11.4 ± 1.2	13.7 ± 1.1	9.9 ± 1.0	7.7 ± 1.0	54.5 ± 1.4
MBG-BRS0231	48.80	0.4 ± 0.8	0.2 ± 0.4	10.0 ± 3.5	15.6 ± 1.9	7.4 ± 1.1	8.2 ± 2.6	54.5 ± 6.8
MBG-BRS0342	51.29	1.5 ± 0.4	0.7 ± 0.1	8.9 ± 1.4	12.7 ± 1.0	10.0 ± 1.3	6.8 ± 1.7	54.4 ± 2.8
MBG-BRS0228	40.38	0.2 ± 0.7	0 ^b^	11.2 ± 2.2	15.4 ± 1.1	8.9 ± 0.8	6.6 ± 0.9	54.3 ± 2.3
MBG-BRS0224	50.26	1.7 ± 1.6	0.5 ± 0.3	9.6 ± 1.0	13.3 ± 1.2	9.5 ± 1.4	6.6 ± 1.3	54.2 ± 4.6
MBG-BRS0181	47.96	1.4 ± 0.5	0.7 ± 0.2	8.9 ± 1.6	14.3 ± 0.9	8.4 ± 1.1	7.5 ± 1.9	54.0 ± 3.3
MBG-BRS0237	47.38	0 ^b^	0 ^b^	10.7 ± 2.4	14.2 ± 1.4	9.8 ± 0.8	8.4 ± 2.5	54.0 ± 3.7
MBG-BRS0244	47.24	2.0 ± 0.2	0.6 ± 0.2	10.4 ± 1.9	11.6 ± 1.0	8.6 ± 0.9	8.2 ± 2.2	54.0 ± 3.2
MBG-BRS0467	50.09	2.3 ± 0.6	0.7 ± 0.2	0.0 ± 1.6	12.5 ± 0.7	8.4 ± 0.5	8.0 ± 1.0	53.8 ± 2.9
MBG-BRS0252	50.89	1.9 ± 0.2	0.6 ± 0.1	10.7 ± 1.3	11.2 ± 0.6	9.7 ± 0.5	8.2 ± 1.1	53.7 ± 2.4
MBG-BRS0349	44.61	2.2 ± 0.3	0.7 ± 0.1	9.3 ± 1.6	13.2 ± 0.7	9.2 ± 0.5	6.7 ± 1.0	53.7 ± 2.9
MBG-BRS0304	44.26	2.0 ± 0.3	0.8 ± 0.3	10.0 ± 1.6	13.0 ± 0.7	8.2 ± 0.5	7.5 ± 1.0	53.6 ± 2.9
MBG-BRS0435	49.13	1.6 ± 0.3	0.8 ± 0.2	9.3 ± 1.6	12.4 ± 0.7	9.8 ± 0.5	7.5 ± 1.0	53.5 ± 2.9
MBG-BRS0101	53.27	2.1 ± 0.3	0.7± 0.1	10.4 ± 1.6	11.9 ± 0.7	8.4 ± 0.5	8.5 ± 1.0	53.5 ± 2.9

^a^ Fatty acids, given as mean ± standard deviation, expressed in % seed oil. ^b^ Values were negligible (only traces). C16:0 = palmitic acid, C18:0 = stearic acid, C18:1= oleic acid, C18:2 = linoleic acid, C18:3= linolenic acid, C20:1= eicosenoic acid, and C22:1 = erucic acid.
